# AI applications across the cancer pain care Continuum: applications and future directions

**DOI:** 10.3389/fpain.2026.1752958

**Published:** 2026-03-18

**Authors:** Danlin Zhu, Han Zhenkai

**Affiliations:** Department of Pain Management, Shengjing Hospital of China Medical University, Shenyang, China

**Keywords:** artificial intelligence, cancer pain, decision support, pain assessment, wearable monitoring

## Abstract

Given the rapid expansion of AI applications across multiple dimensions of cancer pain management, there is an urgent need to synthesize current evidence and clarify future directions to guide clinical and research practice. Cancer pain remains one of the most central and challenging issues in pain medicine. Its pathogenesis is multifaceted, involving tumor invasion, treatment-related injury, and diverse biopsychosocial factors; assessment is difficult, treatment decisions involve numerous variables, and long-term management is resource-intensive. Epidemiological data indicate that approximately half of all patients with cancer experience moderate-to-severe pain during the disease course, with the burden markedly higher in advanced stages. The rapid advancement of AI in medicine has spurred growing interest in its potential contributions to cancer pain assessment, analgesic decision-making, remote follow-up, and interventional planning. This mini-review, organized from a clinical care-pathway perspective, summarizes recent applications of AI in cancer pain assessment, opioid management, remote monitoring, and interventional or longitudinal care. We further discuss methodological and real-world challenges, emphasizing how AI may ultimately contribute to an integrated, longitudinal management framework for cancer pain.

## 1 Introduction

Cancer pain remains highly prevalent, especially among patients with advanced or late-stage malignancies, in whom the proportion experiencing moderate-to-severe pain often approaches or exceeds 60% (1). Its etiology is complex: tumor invasion, inflammatory responses, bone metastasis, nerve injury, and treatment-related factors such as surgery, radiotherapy, and systemic therapy frequently coexist and interact with psychological distress, sleep disturbance, and emotional burden (2). From a pathophysiological perspective, cancer pain arises from heterogeneous and dynamic mechanisms, including nociceptive, neuropathic, and treatment-related processes. These mechanisms are driven by complex interactions among tumor cells, the nervous system, and the surrounding microenvironment, and may evolve across disease stages and therapeutic interventions. As a result, cancer pain often presents with substantial inter-individual variability and mixed phenotypes, posing challenges to uniform assessment and management. This mechanistic heterogeneity provides an important clinical rationale for data-driven approaches, such as artificial intelligence, to integrate multidimensional information across the cancer pain care continuum (3). The clinical pathway for cancer pain typically requires repeated assessment, analgesic adjustment, decisions regarding escalation to interventional or multimodal therapy, management of adverse effects, and both in-hospital and remote follow-up (4). These components are tightly coupled yet highly heterogeneous across individuals.

Traditional decision-making—largely based on clinical experience and linear reasoning—struggles to process the complex variables, hidden patterns, and dynamic trajectories inherent to cancer pain (5). As survival improves and the demand for remote monitoring grows, large amounts of structured and unstructured data are generated in routine cancer care. This increases cognitive workload for clinicians but also creates opportunities for computational approaches. In recent years, AI applications in oncology, anesthesiology, and pain medicine have expanded rapidly (6). Recent studies have applied machine learning and deep learning approaches to multiple aspects of pain care, including automated pain assessment based on behavioral data, as well as clinical decision support for pain-related medication management. In addition, AI-based models have been explored to assist with treatment-related risk stratification and longitudinal pain management in clinical settings (7, 8).

This review follows the clinical logic of “assessment → pharmacologic management → interventional options → follow-up and longitudinal care,” summarizing current progress and discussing future directions. As AI techniques continue to evolve across diverse dimensions of cancer pain management, a focused narrative mini-review of current evidence and emerging directions is needed to guide both clinical practice and future research. Given the heterogeneity of study designs and outcomes in this field, a mini-review format was chosen to provide a concise and clinically oriented overview. Accordingly, this mini-review aims to provide a focused overview of current artificial intelligence applications across the cancer pain care continuum, highlighting their roles in pain assessment, treatment decision-making, and follow-up management, as well as discussing key challenges and future directions for clinical translation.

## 2 AI in cancer pain assessment and phenotype identification

Pain assessment forms the foundation of cancer pain management. Conventional assessment relies heavily on subjective tools such as the Numerical Rating Scale (NRS) and Visual Analogue Scale (VAS), which are constrained by patient comprehension, cooperation, and clinical time. As a result, assessments are often episodic rather than continuous. In recent years, deep learning–driven approaches for more objective pain assessment have emerged, including facial expression analysis and physiological signal monitoring via wearable devices.

Several studies have applied convolutional neural networks (CNNs) to detect pain-related facial features, particularly in children or patients with limited ability to communicate (9, 10). For instance, a CNN-based postoperative pediatric pain expression classifier demonstrated promising accuracy in distinguishing pain from non-pain states in small cohorts, with reported area under the curve values ranging from 0.84 to 0.94 in pediatric postoperative settings (11). This underscores the sensitivity of deep learning in capturing subtle micro-expressions beyond human visual perception (12).

Other studies have used wrist-worn wearable devices to collect physiological signals such as heart-rate variability (HRV) and electrodermal activity (EDA) (13, 14). Machine-learning models—including random forests—trained on these multimodal signals have reliably detected pain episodes in adults with cancer pain. These findings suggest that wearable-based physiological monitoring may complement subjective ratings and offer more continuous, objective assessment. From a functional perspective, physiological biosignals such as HRV and EDA primarily capture autonomic and stress-related responses associated with nociceptive processing, whereas behavioral cues (e.g., facial expression or activity patterns) reflect observable pain-related manifestations. The integration of these complementary signal domains enables a more robust and context-aware assessment than any single modality alone.

Building upon these developments, several groups have applied supervised machine-learning models to classify pain phenotypes or to infer likely pain etiologies based on structured clinical variables, imaging data, and radiotherapy dose–volume information. In this context, pain phenotypes refer to distinct clinical pain presentations (e.g., inflammatory vs. neuropathic pain), whereas pain etiologies describe underlying pathological drivers such as bone metastasis or nerve plexus involvement. Using these input variables, reported models—most commonly tree-based or other supervised classifiers—have demonstrated exploratory capability in identifying clinically relevant pain patterns (15, 16). Although current studies remain exploratory and have limited sample sizes, these models provide a potential technical route toward more refined etiologic reasoning that may support downstream treatment decisions.

Overall, AI applications in cancer pain assessment have expanded from supporting subjective scoring to enabling pain-phenotype recognition, trajectory prediction, and intelligent handling of incomplete longitudinal data. Nonetheless, the field continues to face limitations including small sample sizes, insufficient multimodal integration, and a lack of external validation. Future work should emphasize human–AI collaborative frameworks that integrate clinical information, behavioral markers, wearable-derived physiological signals, and patient-reported outcomes through structured multimodal pathways, enabling hierarchical and temporally informed pain assessment for more granular and individualized evaluation.

## 3 AI-Supported analgesic decision-making and opioid management

Clinically, analgesic adjustments often lag behind the actual worsening of pain, especially in patients undergoing radiotherapy or experiencing rapid changes in disease burden. Thus, proactive prediction is particularly valuable. Machine-learning models integrating demographics, tumor characteristics, radiotherapy parameters, baseline symptoms, and early pain trajectories have shown the ability to predict acute pain peaks and opioid requirements (17). In a cohort of patients with cancer pain, a nomogram based on clinical variables demonstrated high discrimination in predicting analgesic nonadherence, supporting early identification of high-risk patients and timely adjustment of analgesic strategies (18). In our view, these developments signal early signs of a paradigm shift—from episodic subjective assessments to multimodal, continuous, data-driven interpretation—although robust clinical validation remains needed.

Opioids remain the cornerstone of moderate-to-severe cancer pain management. Inadequate analgesia and concerns about nonmedical opioid use or the potential for opioid use disorder (OUD) have received growing attention (19). Multiple studies indicate that opioid-related behaviors among patients with cancer are influenced by pain intensity, breakthrough pain, psychological comorbidities, prior substance use, and patients' attitudes toward opioids (20). Single-scale assessments or clinical intuition alone may not adequately capture this complexity.

Several machine-learning models based on real-world clinical data have attempted to predict the risk of opioid nonadherence. These models incorporate variables such as baseline pain scores, presence of breakthrough pain, psychological assessments, and medication histories, with some achieving area under the curve (AUC) values approaching 0.8 (21). These findings suggest that AI could help identify patients at higher risk of low adherence or unsafe use patterns, enabling earlier counseling, monitoring, and intervention.

Other studies have focused on opioid dosing and opioid-related risk prediction using large-scale electronic health record datasets (22). Multivariable machine-learning models—including random forests and deep learning architectures—have been applied to identify patients with elevated likelihood of opioid-related adverse events or unsafe use patterns. These models have demonstrated promising predictive performance in external datasets, suggesting that early recognition of high-risk individuals may help clinicians avoid reactive “chasing-the-pain” adjustments and unnecessary dose escalation (23). From a clinical perspective, opioid titration often lags behind worsening pain. Therefore, tools that can anticipate analgesic needs or detect emerging risk behaviors may offer greater value than incrementally refining conventional dosing strategies.

## 4 AI-Enabled follow-up and remote management

Cancer pain is a fluctuating and long-term condition, and patients' needs for follow-up intensity vary substantially (24). Some patients maintain stability with infrequent visits, whereas others require close monitoring due to complex pain patterns, frequent breakthrough episodes, or elevated risks associated with opioid therapy. Matching follow-up intensity to clinical need remains a practical challenge.

Machine-learning models have been developed to predict remote-visit demand using real-world databases (25, 26). These models incorporate demographic information, tumor characteristics, pain scores, and prior follow-up patterns. Random forest models have consistently performed well in identifying patients who require closer surveillance, with breakthrough pain and younger age emerging as common indicators of higher follow-up needs.

Other studies have adopted conditional generative adversarial networks (cGANs) to impute missing longitudinal data—such as pain scores—improving model robustness to the incompleteness typical of real-world datasets (27). This is particularly relevant for identifying rare trajectories or patients with irregular visit patterns.

With the growing availability of wearable devices and mobile health platforms, researchers have explored multimodal models combining wrist-worn physiological data (e.g., HRV, EDA), patient-reported outcomes (PROs), and remote video assessments to detect early signs of pain deterioration or the need for medication adjustments (28, 29). This line of work suggests that follow-up scheduling may eventually shift from fixed intervals to risk-driven, individualized monitoring.

Currently, AI applications in follow-up and remote cancer pain management are primarily methodological in nature, constrained by limited integration of multimodal data and a lack of long-term prospective validation. Nevertheless, combining AI with remote monitoring and wearable technology represents one of the most feasible pathways for the digital transformation of cancer pain care.

## 5 From interventional therapy to AI-supported longitudinal care

Interventional therapies—including nerve blocks, neurolytic procedures, celiac or hypogastric plexus ablation, radiofrequency or cryoablation, and intrathecal pump implantation—represent the most “precision-oriented” components of cancer pain management. Although the technical aspects of these procedures are well developed, decisions regarding indications, pathway planning, target localization, and expected duration of benefit still rely on clinical experience, resulting in considerable variability between operators and institutions (30, 31). High-quality evidence and standardized pathways remain limited.

Direct AI research in cancer pain interventions is still scarce. However, evidence from adjacent domains provides insight. In regional anesthesia, deep learning has been used to recognize anatomic structures in ultrasound images, improving the accuracy of needle placement (32, 33). In radiotherapy and tumor ablation, algorithms for automated segmentation of targets and organs-at-risk, as well as optimization of dose distribution, have matured considerably (34). Once adapted to cancer pain scenarios, these techniques could support target selection, needle trajectory planning, complication-risk evaluation, and prediction of post-procedure analgesic duration.

More broadly, pharmacologic and interventional treatments can be viewed as sequential decision nodes within the overall cancer pain pathway. From this perspective, the ideal role of AI is not isolated to individual tasks but embedded within an integrated longitudinal framework. This may involve: early prediction of pain trajectories and analgesic needs; determining when drug therapy is insufficient and interventional escalation is appropriate; identifying patients most likely to benefit from interventions; forecasting duration of analgesia post-procedure; and dynamically adjusting follow-up intensity based on wearable-derived or remote-monitoring data. Although still conceptual, such an integrated approach represents a potential shift from local AI applications toward pathway-level restructuring.

## 6 Discussion

**Figure 1:**
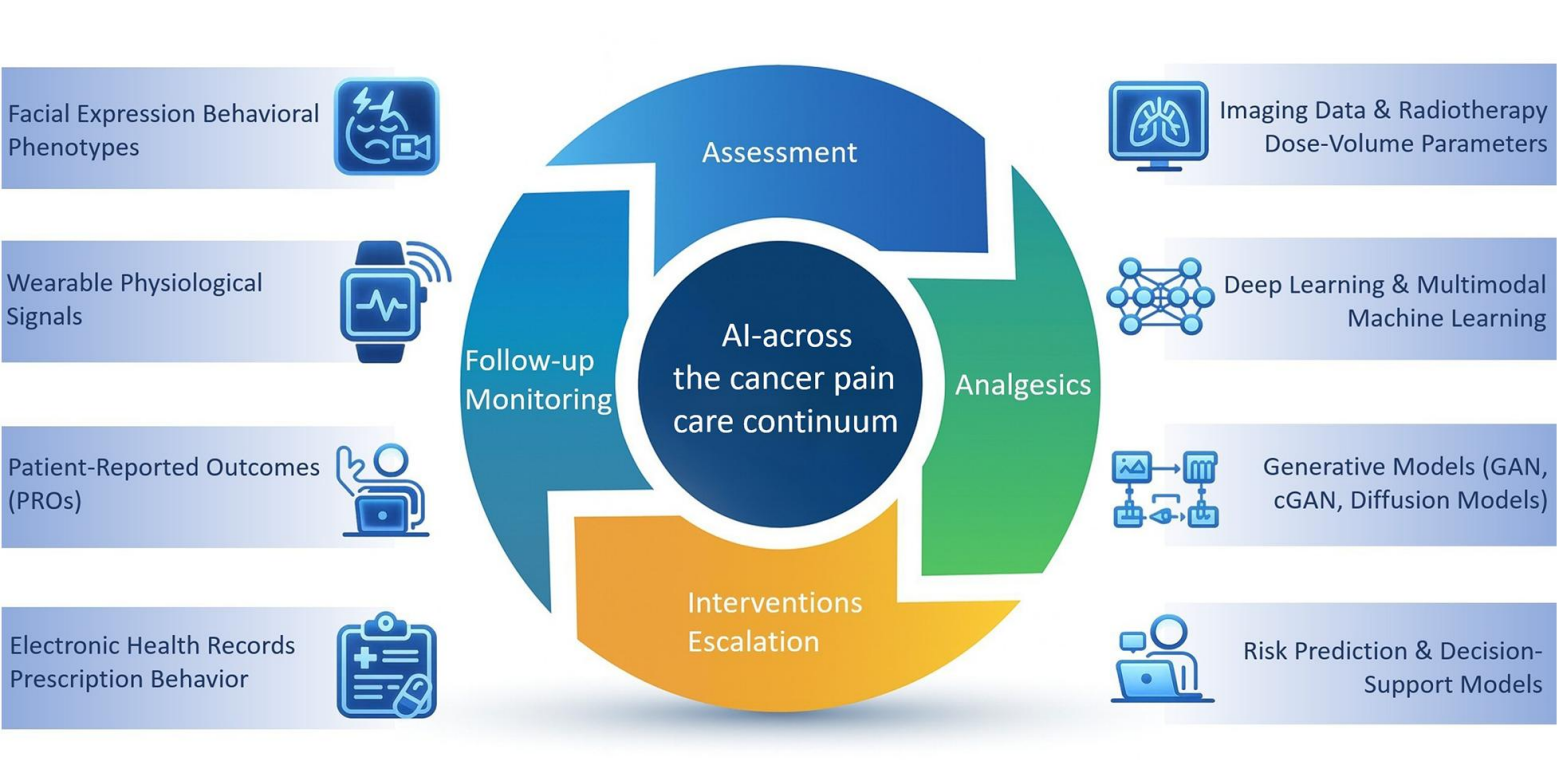
AI-enabled framework illustrating applications of artificial intelligence across the cancer pain care continuum, spanning four key clinical domains and representative data modalities and AI techniques.

Existing literature shows promising AI applications across multiple key areas of the cancer pain care continuum. These include predicting radiotherapy-related pain and opioid requirements; using deep learning and wearable data for pain assessment; identifying risks of opioid nonadherence or misuse; and predicting remote follow-up needs using generative models. Collectively, these developments suggest that AI may enable earlier detection, richer data utilization, and more granular interpretation of pain trajectories. A conceptual summary of these AI-enabled components across the cancer pain care continuum is illustrated in Figure 1.

However, most studies have been limited to single conditions, single centers, or specific technical settings. Moreover, external validation and real-world implementation are still lacking, and the integration of multimodal data and model interpretability is underdeveloped. Cancer pain is a highly heterogeneous condition influenced by biopsychosocial factors, and no single model or data type is likely to provide robust, broadly generalizable decision support. In our view, the primary bottleneck is not model complexity but rather the lack of high-quality, cross-institutional, multimodal datasets. Without reliable data foundations, model performance and generalizability cannot be convincingly established.

Future research should prioritize standardized data collection and multi-center collaboration to build datasets integrating imaging, clinical records, physiological monitoring, psychological assessments, and patient-reported outcomes. Model development should emphasize model interpretability and integration into clinical workflows to ensure usability. Ultimately, embedding pharmacologic management, follow-up scheduling, and interventional decision-making into a unified AI-assisted framework may be key to transforming cancer pain care from isolated tasks to coordinated longitudinal management.

Beyond methodological constraints, practical challenges also limit real-world applications. Cancer pain data span multiple domains, including oncology, pain medicine, imaging, and remote monitoring. Privacy considerations often restrict data sharing between centers. Moreove"r, “black-box” AI models can erode clinician confidence and hinder their integration into clinical decision-making. Patient acceptance of long-term monitoring varies, with concerns regarding data capture and behavioral analysis. Additionally, questions regarding the role of AI in clinical responsibility, workflow integration, and medico-legal boundaries remain unresolved. Addressing these issues will require advances not only in computational methods but also in data governance, the design of interpretable AI systems, and the development of human–AI collaborative workflows.

Despite rapid advances in AI methodologies, several challenges continue to limit real-world translation in cancer pain care. Current studies are frequently constrained by heterogeneous data sources, small or single-center cohorts, and limited external validation, which restrict generalizability across clinical settings. In addition, cancer pain–related data span multiple domains, including oncology, pain medicine, imaging, and remote monitoring, making standardized data integration and governance particularly challenging (35).

Beyond methodological considerations, practical and organizational barriers play a critical role. Concerns regarding data privacy, cross-institutional data sharing, and regulatory compliance often impede large-scale implementation. Moreover, the reliance on “black-box” models may reduce clinician trust and hinder adoption in clinical decision-making, particularly when model outputs lack transparency or explainability. Issues related to workflow integration, clinical responsibility, and medico-legal accountability also remain unresolved. Addressing these challenges will require not only advances in computational techniques, but also the development of human–AI collaborative workflows, clearer governance frameworks, and prospective clinical validation to support safe and effective translation into routine practice. Taken together, this mini-review adopts a pathway-oriented perspective that connects multimodal pain assessment (integrating biosignals and behavioral features) with downstream decision support and longitudinal follow-up across the cancer pain care continuum.

## 7 Conclusion

Artificial intelligence is increasingly being explored as a supportive tool across the cancer pain care continuum, encompassing pain assessment, phenotype characterization, treatment decision-making, and longitudinal management. Available evidence indicates that AI-based approaches may offer added clinical insight and operational support; however, most published studies remain exploratory in nature and are constrained by data heterogeneity, inconsistent outcome definitions, and limited external validation. Future advances will depend on well-designed prospective and multicenter studies, improved model transparency, and closer alignment of AI systems with real-world clinical workflows through effective human–AI collaboration. Addressing these issues will be critical for moving AI beyond proof-of-concept research toward safe, reliable, and clinically meaningful applications in cancer pain care.
